# Gut mycobiome as a potential non-invasive tool in early detection of lung adenocarcinoma: a cross-sectional study

**DOI:** 10.1186/s12916-023-03095-z

**Published:** 2023-10-31

**Authors:** Qingyan Liu, Weidong Zhang, Yanbin Pei, Haitao Tao, Junxun Ma, Rong Li, Fan Zhang, Lijie Wang, Leilei Shen, Yang Liu, Xiaodong Jia, Yi Hu

**Affiliations:** 1 Graduate School, Chinese People’s Liberation Army Medical School, Beijing, China; 2 Department of Oncology, Fifth Medical Center of the Chinese People’s Liberation Army General Hospital, 28 Fuxing Road, Haidian Distrist, Beijing, 100000 China; 3 Department of Thoracic Surgery, First Medical Center of the Chinese People’s Liberation Army General Hospital, 28 Fuxing Road, Haidian District, Beijing, 100000 China; 4 Department of Health Medicine, Second Medical Center of the Chinese People’s Liberation Army General Hospital, Beijing, China; 5 Department of Thoracic Surgery, Hainan Medical Center of the Chinese People’s Liberation Army General Hospital, Hainan, China

**Keywords:** Gut mycobiome, Lung adenocarcinoma, Early-stage diagnosis, Supervised machine learning, Fungal signature, Non-invasive biomarker

## Abstract

**Background:**

The gut mycobiome of patients with lung adenocarcinoma (LUAD) remains unexplored. This study aimed to characterize the gut mycobiome in patients with LUAD and evaluate the potential of gut fungi as non-invasive biomarkers for early diagnosis.

**Methods:**

In total, 299 fecal samples from Beijing, Suzhou, and Hainan were collected prospectively. Using internal transcribed spacer 2 sequencing, we profiled the gut mycobiome. Five supervised machine learning algorithms were trained on fungal signatures to build an optimized prediction model for LUAD in a discovery cohort comprising 105 patients with LUAD and 61 healthy controls (HCs) from Beijing. Validation cohorts from Beijing, Suzhou, and Hainan comprising 44, 17, and 15 patients with LUAD and 26, 19, and 12 HCs, respectively, were used to evaluate efficacy.

**Results:**

Fungal biodiversity and richness increased in patients with LUAD. At the phylum level, the abundance of Ascomycota decreased, while that of Basidiomycota increased in patients with LUAD. *Candida* and *Saccharomyces* were the dominant genera, with a reduction in *Candida* and an increase in *Saccharomyces*, *Aspergillus*, and *Apiotrichum* in patients with LUAD. Nineteen operational taxonomic unit markers were selected, and excellent performance in predicting LUAD was achieved (area under the curve (AUC) = 0.9350) using a random forest model with outcomes superior to those of four other algorithms. The AUCs of the Beijing, Suzhou, and Hainan validation cohorts were 0.9538, 0.9628, and 0.8833, respectively.

**Conclusions:**

For the first time, the gut fungal profiles of patients with LUAD were shown to represent potential non-invasive biomarkers for early-stage diagnosis.

**Supplementary Information:**

The online version contains supplementary material available at 10.1186/s12916-023-03095-z.

## Background

Lung cancer remains the leading cause of cancer-related deaths and a major public health issue worldwide. Non-small cell lung cancer (NSCLC) accounts for approximately 85% of all lung cancer cases. Adenocarcinoma is the most common histological subtype of NSCLC [1, 2]. Five-year survival rates in patients with lung cancer are heavily influenced by the disease stage at diagnosis. Patients diagnosed with distant metastatic tumors (stage IV) have a 5-year survival rate of only 5.2% compared with 57.4% for small, localized tumors (stage I). Despite advances in detection and treatment, approximately 57% of patients are still initially diagnosed at an advanced stage (stage III/IV) with a poor prognosis [3], and predictive biomarkers for early detection remain unsatisfactory. Thus, exploring novel early diagnostic markers is warranted to prompt early intervention and improve long-term outcomes.

The gut microbiome profoundly influences human health and is involved in multiple chronic disorders [4–6]. The interaction between gut microbiota and the lung, the “gut–lung axis,” has been extensively studied, although the mechanisms by which the gut microbiota affects lung immunity are still unclear [7, 8]. Gut microbial dysbiosis has been linked to a number of lung diseases and disorders, including asthma and chronic obstructive pulmonary disease [9, 10]. Both human epidemiological evidence and animal studies suggest that early-lifetime dysbiosis of gut microbiota increases the risk of allergic respiratory diseases [11–14]. Although the interactions between microbiota dysbiosis and cancer development and progression as well as cancer therapy has been extensively studied [15–17], research to date has mainly focused on bacteria, whereas fungi have largely been overlooked due to their relatively low abundance (less than 0.1% of all microorganisms in the gut) as well as a lack of well-characterized reference genomes [18, 19], meaning that new diagnostic and preventive strategies are not being pursued.

The fungal microbiome plays an important role in maintaining intestinal homeostasis and the host immune system despite the low abundance of fungi [18]. As a consequence of the significant technological development in bioinformatics methodologies, fungi populating the human gut are increasingly being identified and an increasing number of studies have provided insights into the association between gut fungi and different diseases [20, 21]. Cancer–mycobiome interactions have recently attracted considerable interest, as alterations in gut fungi are specific to different cancer types. In patients with colorectal cancer, an alteration in the gut mycobiome includes decreases in Saccharomycetes and Pneumocystidomycetes and the enrichment of Malasseziomycetes [19, 22]. Fungal abundance in patients with pancreatic ductal adenocarcinoma (PDAD) undergoes a more than 3000-fold increase compared with that in healthy controls. *Malassezia* spp. are abundant in patients with PDAD, and its enrichment accelerates tumor growth [23, 24]. In addition, it has been established that specific tumor tissues are characterized by distinct fungal DNA profiles. For example, high levels of *Candida* are detected in gastrointestinal cancer tissues and are predictive of poor survival [25]. Thus, fungal dysbiosis plays a pivotal role in the pathogenesis, progression, and prognosis of cancer and might serve as a non-invasive diagnostic or prognostic biomarker. To our knowledge, no studies have been conducted to characterize the gut mycobiome of patients with LUAD, particularly from the perspective of using fungal signatures as non-invasive diagnostic biomarkers.

Machine learning (ML) technology, a powerful tool that can process vast amounts of data, has been widely used in cancer medicine and shows excellent performance with high accuracy in the predictive and diagnostic fields [26, 27]. Supervised and unsupervised algorithms are the main types of ML applied, the former being the most widely adopted in analysis of the gut microbiome, performed with a view toward identifying microbial biomarkers for prediction of disease risk [28].

Here, we characterized the gut mycobiomes of patients with LUAD using internal transcribed spacer (ITS) 2 sequencing and applied ML technology to construct a diagnostic model for early-stage LUAD based on selected operational taxonomic units (OTUs). Considering that the mycobiome composition is influenced by multiple factors, including gender, age, diet, lifestyle, medication (antibiotics or immunosuppressive drugs), and geography [29], validation cohorts from different regions in China were used to evaluate the utility of the gut fungal signature as a non-invasive biomarker while minimizing the influence of confounding factors.

## Methods

### Study design

**Fig. 1:**
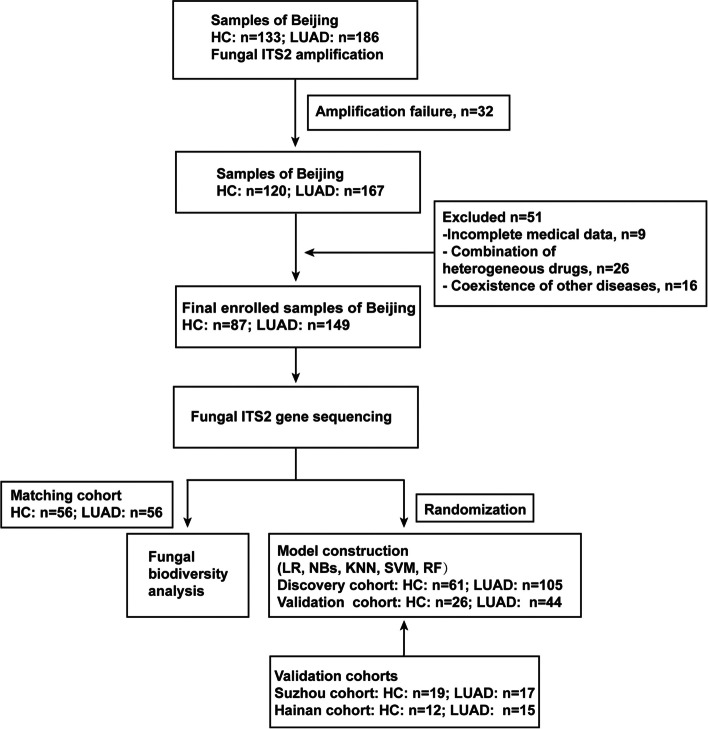
Study design and flowchart. We enrolled 181 patients with LUAD and 118 HCs from Beijing, Suzhou, and Hainan and prospectively collected fecal samples for ITS 2 sequencing. We characterized fungal biodiversity in the matching cohort from Beijing. OTU markers were obtained in the discovery cohort and established a prediction model for LUAD using five supervised ML algorithms. Validation cohorts from Suzhou and Hainan were used to evaluate efficacy

In total, 299 participants, comprising 181 patients with LUAD and 118 healthy controls (HCs) from Beijing, Suzhou, and Hainan, were recruited. The discovery cohort comprised of 105 patients with LUAD and 61 HC participants from Beijing. The internal validation cohort comprised of 44 patients with LUAD and 26 HCs from Beijing. External validation cohort 1 from Suzhou comprised 17 patients with LUAD and 19 HCs. External validation cohort 2 from Hainan comprised 15 patients with LUAD and 12 HCs. Samples from Beijing were randomly divided into a discovery cohort and an internal validation cohort (7:3) using the R software [30]. The analysis of gut mycobiome diversity and composition was conducted in matching cohort of 56 patients with LUAD and 56 HCs among the Beijing participants matched for age, gender, and BMI. The generation of matching cohort was performed by Propensity Score in the R project Nonrandom package (version 1.1), to control for confounding factors among the statistical age, gender, and BMI differences between the two groups (Fig. 1). The key inclusion criteria were as follows: (1) age ≥ 18 years, (2) pathologically diagnosed with LUAD by the surgical specimen, (3) a diagnosis of LUAD at pathological stage II or earlier, and (4) provision of informed consent. Patients were excluded if they (1) presented with other pathological types of lung cancer or other malignancies or with a previously diagnosed malignancy, (2) had been administered antibiotics or probiotics within eight weeks before the study, (3) had undergone chemotherapy, immunotherapy, or any traditional Chinese medicinal treatments, or (4) provided incomplete information. The demographic data and clinical characteristics of patients were obtained from the patients’ electronic medical records or patient descriptions based on direct interviews or follow-up by telephone. Basic information pertaining to the participants in different cohorts are presented in Table S1 (Additional file 1: Table S1) and Table S2 (Additional file 1: Table S2). The study was approved by the Ethics Committee of the Chinese People’s Liberation Army General Hospital (S2022-407–01).

### ITS amplification and bioinformatics analysis

Stool samples were collected prospectively in sterile boxes, transported to the laboratory on ice, and then stored at − 80 °C. DNA was extracted using HiPure Stool DNA Kits (Magen, Guangzhou, China) according to the manufacturer’s instructions. DNA content and purity were assessed using a NanoDrop 2000 (Thermo Fisher Scientific, Waltham, MA, USA). DNA was stored at − 20 °C until use. Fungal ITS2 amplification was performed using the primers ITS3_KYO2 (5′-GATGAAGAACGYAGYRAA-3′) and ITS4 (5′-TCCTCCGCTTATTGATATGC-3′) [31]. PCR amplification of genomic DNA was performed using 20-μL reaction mixtures under the following conditions: an initial denaturation at 95 °C for 5 min, followed by 33 cycles at 95 °C for 1 min, 60 °C for 1 min, and 56 °C for 1 min, and a final extension at 72 °C for 7 min (Additional file 2: Table S3). The amplicons were run on 2% agarose gels and purified using an AxyPrep DNA Gel Extraction Kit (Axygen Biosciences, Tewksbury, MA, USA) prior to library pooling and quantified using a StepOnePlus Real-Time PCR System (Thermo Fisher Scientific). The purified amplicons were then subjected to a second round PCR using 50-μL reaction mixtures under the following conditions: 95 °C for 5 min, followed by 12 cycles of 95 °C for 1 min, 60 °C for 1 min, and 72°C for 1 min, and a final extension at 72°C for 7 min (Additional file 2: Table S4). Purified amplicons were pooled at equimolar concentrations and paired-end sequenced (read type PE250) on the Illumina NovaSeq platform (Illumina, San Diego, CA, USA) using standard protocols.

Chao1 and Shannon indices were calculated using QIIME [32] (version 1.9.1). Alpha index comparison between groups was calculated using the Wilcoxon rank test in the R project Vegan package (version 2.5.3). Principal coordinate analysis (PCoA) of the Bray–Curtis distance and Adonis statistical analysis were carried out in the R project Vegan package (version 2.5.3) and plotted in the R project ggplot2 package (version 2.2.1). The Wilcoxon rank-sum test was used to identify differences in the mycobiomes of the HC and LUAD groups.

### OTU-based construction of machine learning models

UPARSE [33] (version 9.2.64) was used to cluster OTUs with a 97% similarity cut-off value into the same operational classification unit. OTU features selected with the Boruta package (version 8.0.0) in R were used for ML model construction. To construct a prediction model for early-stage LUAD, the mlr3 R package (version 0.14.1) was used for five common ML algorithms: random forest (RF), k-nearest neighbors (KNN), naïve Bayes (NBs), support vector machine (SVM), and logistic regression (LR). The best model was determined by comparing the accuracy of the five ML algorithms. A classification error loss function (“ce”) in the R package “iml” (version 0.11.1) was used to calculate the importance of 19 OTUs.

### Statistical analysis

SPSS 25.0 software (IBM, Armonk, NY, USA) was used for the statistical analysis of basic information of participants. A two-sided chi-square test or Fisher’s exact test was used to compare categorical variables between the two groups, whereas a two-sided *t*-test was used for normally distributed continuous variables, and a two-sided Wilcoxon rank-sum test was used for non-normally distributed continuous variables. A 1:1 propensity score-matched pair method combined with covariate adjustment was used to balance the unbalanced baseline conditions of the matching cohort, resulting in matched pairs with no difference in age, gender and BMI. *P*-value < 0.05 was considered significant.

## Results

### Diversity of gut fungi in patients with LUAD compared with healthy controls

**Fig. 2:**
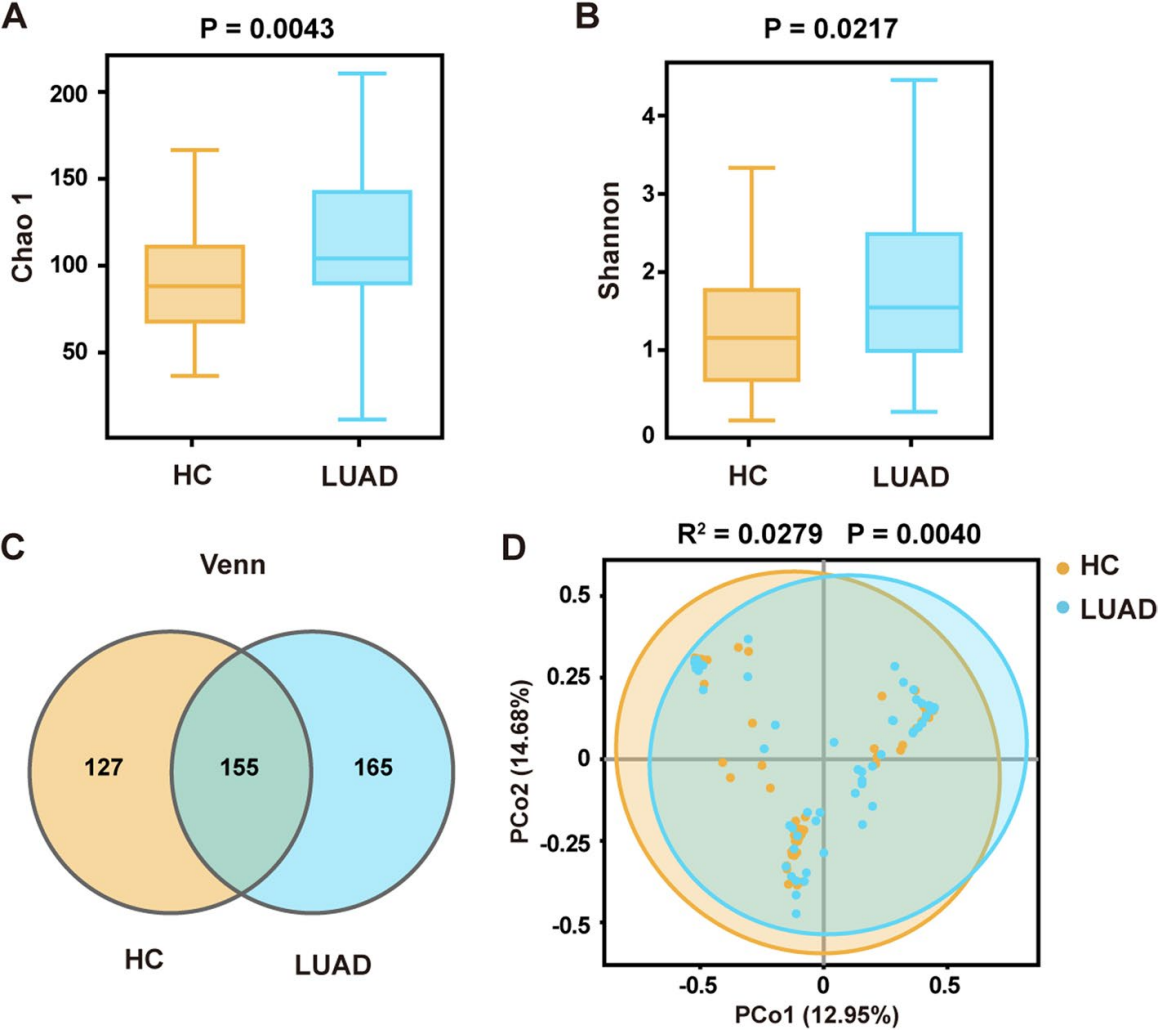
Changes in fungal biodiversity in LUAD. **A**, **B** Alpha diversity. Chao1 and Shannon indices describe the α-diversity of the fungi in the LUAD and HC groups. Relative to results in controls, the α-diversity was increased significantly in patients with LUAD (*P* = 0,0043 and *P* = 0.0217). **C** Venn diagram analysis of OTU abundance between the two groups. The overlap shows that 155 OTUs were shared between the two groups, while 165 and 127 OTUs were unique for in the LUAD and HC groups, respectively. **D** Beta diversity. Principal coordinate analysis of Bray–Curtis distance with each sample colored by group. Groups were compared using the Adonis method (Adonis *R*.^2^ = 0.0279, *P* = 0.0040)

Differences in fungal richness and diversity between the LUAD and HC groups were assessed. The α-diversity was evaluated using Chao1 (Fig. 2A) and Shannon indices (Fig. 2B), and the results showed that biodiversity in the LUAD group was significantly higher than that in the HC group (Chao1, *P* = 0.0043; Shannon index, *P* = 0.0217). A Venn diagram is used to show the distribution of common and endemic OTUs between the two groups based on OTU abundance (Fig. 2C). A total of 155 OTUs were shared between the two groups, with 165 and 127 OTUs unique to the LUAD and HC groups, respectively. The number of OTUs in the LUAD group was higher than that in the HC group, indicating that the fungal diversity in patients with LUAD was higher than that in HCs. The β-diversity was used to evaluate differences in microbial community compositions between the two groups using the Bray–Curtis distance (Fig. 2D). PCoA showed that individuals in the LUAD group were well distinguished from those in the HC group, demonstrating that the fungal communities in the two groups were considerably different (Adonis *R*^2^ = 0.0279, *P* = 0.0040).

### Gut mycobiome composition at phylum and genus level

**Fig. 3:**
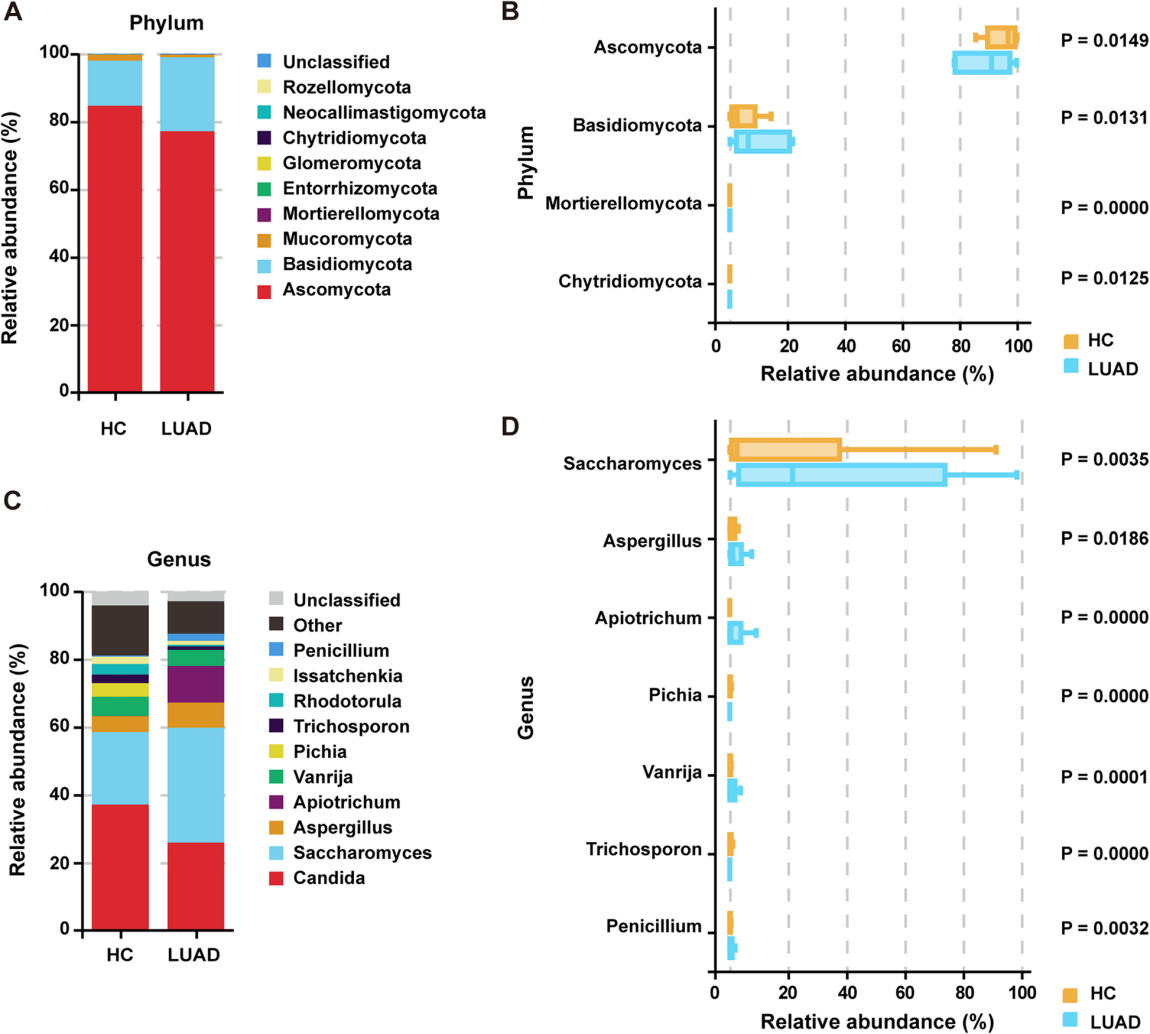
Changes in the gut fungal composition. Differential abundance of dominant fungal taxa at the phylum (**A**–**B**) and genus (**C**–**D**) level between the LUAD and HC groups (Wilcoxon rank-sum test, the respective *P*-values are shown in the diagram)

The mycobiome composition was evaluated at various taxonomic levels. Considerable variations in abundance at both the phylum and genus levels were observed between the two groups. Overall, Ascomycota and Basidiomycota were the most predominant phyla (Fig. 3A). Compared with the HC group, the LUAD group showed a lower abundance of Ascomycota (*P* = 0.0149) and significantly higher abundances of Basidiomycota (*P* = 0.0131), Mortierellomycota (*P* = 0.0000), and Chytridiomycota (*P* = 0.0125) (Fig. 3B). At the genus level, *Candida* and *Saccharomyces* were the most abundant genera in both groups. The abundance of *Saccharomyces* (*P* = 0.0035), *Aspergillus* (*P* = 0.0186), *Apiotrichum* (*P* = 0.0000), and *Penicillium* (*P* = 0.0032) were dramatically elevated in patients with LUAD compared with that in HCs. Conversely, the proportion of *Vanrija* (*P* = 0.0001), *Pichia* (*P* = 0.0000), and *Trichosporon* (*P* = 0.0000) were markedly lower in the LUAD group (Fig. 3C, D).

### Identification of a gut fungal OTU-based signature of early LUAD

**Fig. 4:**
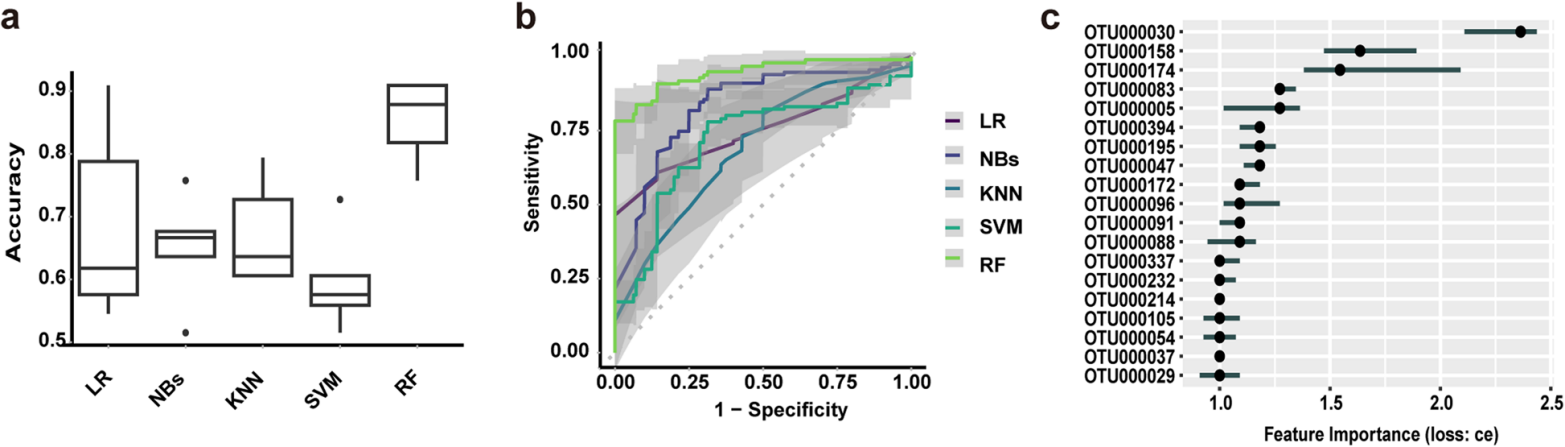
Identification of fungal OTU-based signatures of early-stage LUAD. The Boruta algorithm was first used to select 19 OTUs as the final features, and five different supervised ML algorithms were used for identifying patients with LUAD based on OTU features in the discovery cohort. **A**, **B** Accuracy performance (**A**) and receiver operating characteristic curves (**B**) of LR, NBs, KNN, SVM, and RF algorithms. RF achieved the highest accuracy of 86.17% and the maximum AUC of 0.9350. **C** Importance of the 19 OTU features was ranked using a classification error loss function

**Fig. 5:**
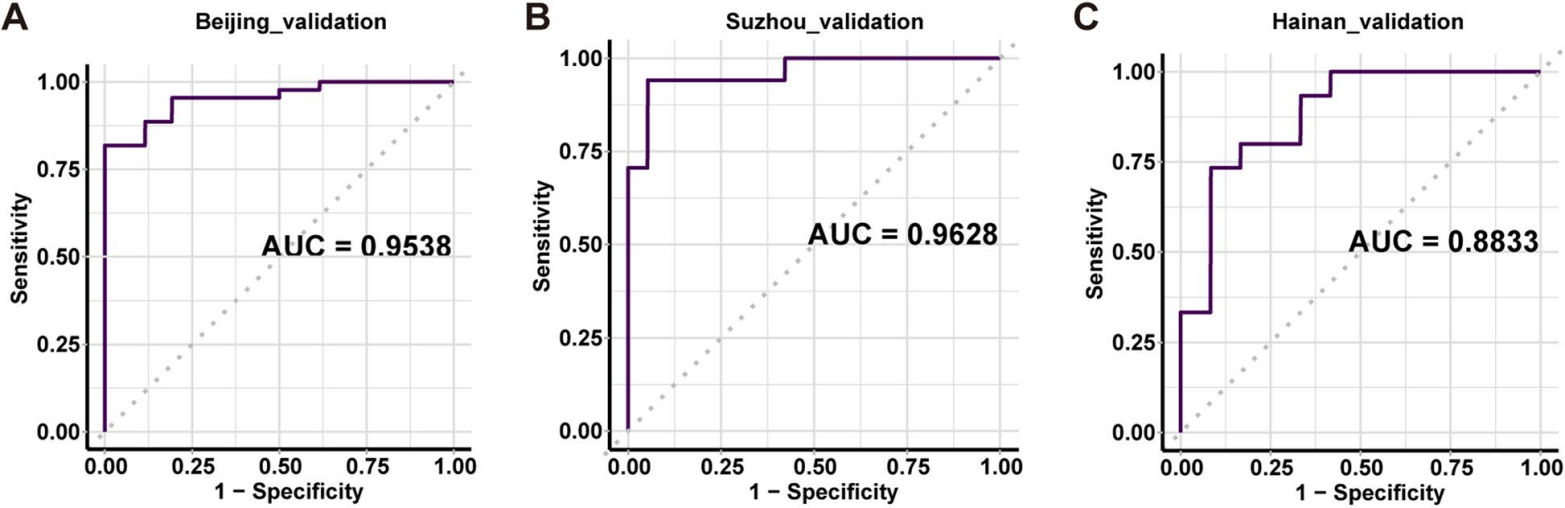
Validation of the selected OTU features for LUAD. **A** The OTU features gave an AUC for the ROC curve of 0.9538 using RF with the internal validation cohort. **B**, **C** The OTU features gave AUCs of 0.9628 and 0.8833, respectively, using RF with the external validation cohorts from Suzhou and Hainan

Clinically, the development of non-invasive diagnostic biomarkers for early-stage lung cancer is of key importance. In this regard, we used the Beijing discovery cohort for OTU selection and ML model construction. The Boruta algorithm was used to select 19 OTUs as final features for model construction, and five supervised ML algorithms were trained using these OTUs. Differences of the abundances of these 19 OTUs between the LUAD and HC groups are shown in Fig. S1 (Additional file 3: Fig. S1). Figure 4A and B present results from the five ML models evaluated in the discovery cohort. Of all ML models, RF had the best performance and achieved the highest accuracy of 86.17% in classifying LUAD and HC individuals, with less accurate results seen using KNN (69.32%), LR (65.00%), SVM (61.98%), or NB (60.87%). In terms of the training area under the curve (AUC), RF performed the best with AUC values of up to 0.9350 (95% CI: 0.8933–0.9766), while the corresponding AUC values were lower for KNN (0.6878, 95% CI: 0.5923–0.7833), LR (0.5973, 95% CI: 0.3287–0.8660), SVM (0.6810, 95% CI: 0.5634–0.7986), and NB (0.7099, 95% CI: 0.5627–0.8571). Overall, RF produced results superior to those of the other ML models in predicting LUAD. We used a classification error loss function (“ce”) to calculate the importance of the 19 OTUs. OTU000030 and OTU000158 were the two most critical features predicting LUAD in the training model (Fig. 4C). In the internal validation phase, RF had high predictive power with an AUC of 0.9538 (95% CI: 0.9063–1) (Fig. 5A). Moreover, we found that the RF model showed a good performance when applied to the matching cohort (Additional file 3: Fig. S2). To further confirm the diagnostic potential of the OTU markers in other samples, two external validation cohorts from Suzhou and Hainan were used for independent testing to confirm the reliability of RF. The Suzhou validation cohort had a surprisingly high AUC value of 0.9628 (95% CI: 0.8963–1), while the Hainan validation cohort had an AUC value of 0.8833 (95% CI: 0.7539–1), slightly lower than that in the discovery cohort (Fig. 5B, C). The data show that the fungal OTU markers possessed a strong diagnostic classification efficacy for patients with early-stage LUAD from northern and southern China.

## Discussion

Our study represents the first characterization of the gut mycobiome composition of a large cohort of patients with early-stage LUAD. Additionally, we presented an innovative and non-invasive approach involving gut mycobiome-based ML classification for the convenient diagnostic screening of LUAD. A diagnostic model based on microbial OTU markers was successfully established and validated across three different regions in China.

To date, studies on the gut mycobiome have been limited to varying extents by a deficiency of appropriate detection methods (i.e., fungi are less amenable to culturing than bacteria), technical limitations, and a lack of comprehensive reference databases. However, the rapid advances in bioinformatics analysis methodology in recent years have facilitated an acceleration in the identification of fungi, which is expanding our knowledge of the fungal kingdom and the contribution of fungi to human health and disease. With respect to high-throughput sequencing, the selection of appropriate barcoding primers and amplification conditions is considered a key prerequisite. In this context, amplification and sequencing of the ITS1 (between 18S and 5.8S) and ITS2 (between 5.8S and 28S) regions is a widely adopted approach in studies of the human gut mycobiome [21], although a consensus has yet to be reached regarding the selection of ITS sub-regions. On the basis of a survey of the relevant literature, it would appear that compared with ITS1, ITS2 is associated with less amplification and sequencing bias [21, 34]. Consistent with this assessment, in a preliminary phase of this study, we had relatively limited success when using primers targeting ITS1 [31]. Consequently, on the basis of these findings, in the present study, we selected primers targeting the ITS2 sub-region.

Fungi are complex organisms known to play an opportunistic role during immunosuppressive and antibiotic therapies [18]. Fungal invasion induces the synthesis of various signaling molecules, including transforming growth factor-β, interleukin (IL)-6, IL-12, IL-23, IL-1β, and interferon-γ, which trigger Th1 and Th17 cell responses, in parallel with macrophage activation and neutrophil recruitment [18, 35]. Inflammation induced by pathogens is a major mechanism promoting carcinogenesis [36]. The promotion of carcinogenesis by fungal metabolites has been suggested as another major mechanism. The carcinogenic effects of acetaldehyde [37] produced by *Candida* and aflatoxin [38] produced by *Aspergillus* have been demonstrated. In our study, the intestinal fungal profiles of LUAD cases differed from those of HCs. The gut fungal diversity and richness markedly increased during the progression of LUAD, suggesting that mycobiome alterations potentially promote the pathological progression of LUAD. The predominant phyla in both patients with LUAD and HCs were Ascomycota and Basidiomycota, consistent with previously reported fungal profiles in other malignant tumor types [39]. The abnormal changes in the abundance of Ascomycota and Basidiomycota in the LUAD group may reflect fungal dysbiosis, in line with prior reported studies on colorectal cancer and pancreatic cancer [22, 23]. At the genus level, *Candida* and *Saccharomyces* were the most abundant in our cohort. Previous studies have shown that *Candida*, *Saccharomyces*, *Malassezia*, and *Cladosporium* spp. are the most prevalent fungi in the healthy human gut [40]. However, slight variations in the dominant genera are found in different study cohorts, possibly due to sample size bias or the different geographical locations of participants. Hoffman et al. [41] have reported that *Saccharomyces*, *Candida*, and *Cladosporium* are the most abundant genera in healthy subjects. In a study by Nash et al. [42], *Saccharomyces*, *Malassezia,* and *Candida* were the most abundant genera in healthy subjects. *Candida* is a prominent opportunistic fungal pathogen in humans and is involved in many other diseases, including inflammatory bowel disease (IBD) [43, 44], alcohol-associated liver disease [45, 46], asthma [47], and COVID-19 [48]. A recent study on pan-cancer mycobiomes in tumor tissues has revealed that *Candida* is associated with pro-inflammatory gene expression, tumor metastasis, and poorer survival outcomes, especially for gastrointestinal cancers, indicating that the detection of *Candida* may represent a novel predictive biomarker and therapeutic target [25]. Although *Candida* was the most predominant genus in this study, it was not associated with the disease phenotype. In contrast, the proportion of *Saccharomyces* was significantly higher in patients with LUAD than in controls. *Saccharomyces* spp., as “bakers” and “brewers” yeasts, are commonly used in food fermentation. The role of *Saccharomyces* in disease is controversial. Saumya et al. [49] have identified *Saccharomyces* as the most abundant (42%) genus in patients with multiple sclerosis (MS), a chronic autoimmune disease of uncertain etiology. In addition to the increase in *Saccharomyces* in patients with MS compared with the controls, it is also associated with the peripheral immune response, implying a pathogenic correlation between *Saccharomyces* and MS. In contrast, Harry et al. [44] have reported that *Saccharomyces* and especially *Saccharomyces cerevisiae* show a markedly decreased abundance in patients with IBD, whereas *S. cerevisiae* exhibits anti-inflammatory effects involving increased secretion of IL-10. These results highlight the complexity of fungi–host interactions and the urgent need for the further exploration of their effects on health and disease.

As the gut mycobiome is a highly variable and dynamic community, limited sample sizes for disease-associated fungal taxa may not be reliable biomarkers in diagnostic applications. Therefore, in addition to analyzing changes in gut fungal composition in patients with early-stage LUAD, our study applied OTU-based gut mycobiome features to train a supervised ML model. ML refers to a wide range of algorithms that can make predictions that mimic human decisions and represents a major form of artificial intelligence [50]. Cutting-edge computer technologies of this kind have been widely used in the healthcare field and have achieved remarkable results, such as the use of artificial intelligence image recognition technology to diagnose multiple malignant tumor patients accurately through medical images [51–53] and the use of ML to predict the prognosis and survival of patients with malignant tumors [27, 54]. However, some uncertainty exists about the diagnostic efficacy [55]. In our study, an exploratory analysis of five commonly available supervised ML algorithms was carried out to compare the performance in predicting LUAD. The results showed that RF achieved an excellent predictive AUC of 0.9350 for distinguishing patients with early-stage LUAD from healthy subjects. Moreover, considering that gut microbiota may be influenced by diet and geography, we conducted cross-regional validation to better verify the efficacy and applicability of the models. Similar to gut bacteria, the gut mycobiome undergoes changes during the human lifetime, and the geography, dietary habits, and host factors, including sex, age, and drug use, are prominent factors that contribute to shaping the gut mycobiome composition [56]. Yang et al. characterized gut mycobiome profiles across different regions in China, including six ethnicities at a large population scale, and accordingly found that geography and ethnicity have pronounced effects on the variations in gut fungi [57]. In the present study, despite the confounding factors of geography and diet, all the validation cohorts showed excellent results, thereby indicating the potential significance of fungal markers in the diagnosis of LUAD and the broad applicability of our approach in different geographical regions.

The limitations of the current study include the low number of fecal samples from the Suzhou and Hainan cohorts. Self-reported drug intake may introduce a certain degree of bias. A larger sample size and stricter screening criteria in multiple centers are needed to further validate the results. In addition, further animal studies are required to verify the potential association between altered fungal diversity and tumor formation.

## Conclusions

We elucidated the characteristic gut fungal alterations in patients with LUAD in a large clinical cohort, screened OTU-based fungal markers, and applied supervised ML models to validate the diagnostic efficacy in cohorts from different regions in China. Notably, despite the possibility of misdiagnosis, our study demonstrates the potential of training supervised ML models using intestinal fungal factors for the clinical diagnosis of LUAD. We hope to better assist the development of diagnostic and therapeutic targets in lung cancer and further benefit patients.

### Supplementary Information

**Additional file 1. **Basic information of the participants. **Table S1.** Baseline characteristics of all Beijing participants and the matching cohort. **Table S2.** Baseline characteristics of the discovery and validation cohorts.

**Additional file 2. **The PCR amplification conditions. **Table S3.** The first round PCR system. **Table S4.** The second round PCR system.

**Additional file 3. **Supplementary figures. **Fig. S1.** Abundance differences of the 19 OTUs between the LUAD and HC groups in the Beijing discovery cohort. All of the 19 OTUs are significant different in the two groups (Wilcoxon rank-sum test, the respective *P*-values are shown in the diagram). **Fig. S2.** AUC of the selected OTU features for LUAD validated in the matching cohort.

## Data Availability

The raw sequence data reported in this paper have been deposited in the Genome Sequence Archive in National Genomics Data Center, China National Center for Bioinformation/Beijing Institute of Genomics, and Chinese Academy of Sciences (GSA-Human: HRA004795) that are publicly accessible at https://ngdc.cncb.ac.cn/gsa-human.

## Abbreviations

AUC: Area under the curve
BMI: Body mass index
HC: Healthy control
IBD: Inflammatory bowel disease
IL: Interleukin
ITS: Internal transcribed spacer
KNN: K-Nearest neighbors
LR: Logistic regression
LUAD: Lung adenocarcinoma
ML: Machine learning
MS: Multiple sclerosis
NB: Naïve Bayes
NSCLC: Non-small cell lung cancer
OTU: Operational taxonomic units
PCoA: Principal coordinate analysis
PDAD: Pancreatic ductal adenocarcinoma
RF: Random forest
SVM: Support vector machine
