# Supplementary material for: Gut mycobiome as a potential non-invasive tool in early detection of lung adenocarcinoma: a cross-sectional study
Source: BMC Med. 2023 Oct 31;21:409. doi: 10.1186/s12916-023-03095-z (PMC10617124; doi:10.1186/s12916-023-03095-z)
Supplement: Supplementary file 1 — Additional file 1. Basic information of the participants. Table S1. Baseline characteristics of all Beijing participants and the matching cohort. Table S2. Baseline characteristics of the discovery and validation cohorts. [file 12916_2023_3095_MOESM1_ESM.docx]

**Table S1.** Baseline characteristics of all Beijing participants and the matching cohort.

| Cohort | Factor | Total | HC group | LUAD group | P values |
| --- | --- | --- | --- | --- | --- |
| All Beijing  participants | Sample size | 236 | 87 | 149 |  |
|  | Age | 54.66±10.55 | 51.33±8.92 | 56.60±10.96 | <0.001 |
|  | Gender |  |  |  | <0.001 |
|  | Male | 120 | 66 | 54 |  |
|  | Female | 116 | 21 | 95 |  |
|  | BMI | 24.56±3.19 | 25.70±3.15 | 23.89±3.02 | <0.001 |
|  | Tumor size |  |  |  |  |
|  | ≤3cm | 148 | / | 148 | / |
|  | 3-5cm | 1 | / | 1 | / |
|  | N stage |  |  |  |  |
|  | N_0_ | 145 | / | 145 | / |
|  | N_1_ | 4 | / | 4 | / |
|  | Tumor stage |  |  |  |  |
|  | Ⅰ |  | / | 144 | / |
|  | Ⅱ |  | / | 5 | / |
|  | CEA (0-5 μg/L) | 1.10(0.73-1.66) | 0.80(0.70-1.00) | 1.37(0.91-2.25) | <0.001 |
|  | CYFRA21-1 (0.1-4.0 ng/ml) | 3.86(3.12-5.01) | 3.45(2.41-4.69) | 4.06(3.41-5.03) | <0.001 |
|  | SCC (<1.8 ng/ml) | 1.40(1.11-1.80) | 1.40(1.04-1.88) | 1.40(1.20-1.70) | 0.607 |
|  | NSE (0-24 ng/ml) | 9.68(3.79-11.81) | 1.89(1.13-4.70) | 11.17(9.72-12.68) | <0.001 |
| Matching cohort (Beijing) | Sample size | 112 | 56 | 56 |  |
|  | Age | 53.36±10.01 | 53.96±9.00 | 52.75±10.98 | 0.523 |
|  | Gender |  |  |  | 0.699 |
|  | Male | 69 | 35 | 34 |  |
|  | Female | 43 | 21 | 22 |  |
|  | BMI | 24.81±3.18 | 24.88±3.23 | 24.75±3.16 | 0.827 |
|  | Tumor size |  |  |  |  |
|  | ≤3cm | 56 | / | 56 | / |
|  | 3-5cm | 0 | / | 0 | / |
|  | N stage |  |  |  |  |
|  | N_0_ | 54 | / | 54 | / |
|  | N_1_ | 2 | / | 2 | / |
|  | Tumor stage |  |  |  |  |
|  | Ⅰ | 54 | / | 54 | / |
|  | Ⅱ | 2 | / | 2 | / |
|  | CEA (0-5 μg/L) | 1.10(0.70-1.62) | 0.80(0.60-1.00) | 1.54(1.16-2.36) | <0.001 |
|  | CYFRA21-1 (0.1-4.0 ng/ml) | 3.69(3.01-4.82) | 3.42(2.35-4.56) | 3.90(3.33-4.56) | <0.001 |
|  | SCC (<1.8 ng/ml) | 1.40(1.11-1.77) | 1.43(1.05-1.82) | 1.40(1.20-1.60) | 0.762 |
|  | NSE (0-24 ng/ml) | 7.55(1.84-11.03) | 1.85(1.32-4.14) | 10.84(9.40-12.33) | <0.001 |

**Table S2.** Baseline characteristics of the discovery and validation cohorts.

| Cohort | Factor | Total | HC group | LUAD group | P values | |
| --- | --- | --- | --- | --- | --- | --- |
| Training  cohort（Beijing） | Sample size | 166 | 61 | 105 | |  |
|  | Age | 54.04±10.40 | 50.05±7.34 | 56.49±11.21 | | ＜0.001 |
|  | Gender |  |  |  | | ＜0.001 |
|  | Male | 81 | 45 | 36 | |  |
|  | Female | 85 | 16 | 69 | |  |
|  | BMI | 24.43±3.07 | 25.54±2.93 | 23.78±2.97 | | ＜0.001 |
|  | Tumor size |  |  |  | |  |
|  | ≤3cm | 104 | / | 104 | | / |
|  | 3-5cm | 1 | / | 1 | | / |
|  | N stage |  |  |  | |  |
|  | N_0_ | 102 | / | 102 | | / |
|  | N_1_ | 3 | / | 3 | | / |
|  | Tumor stage |  |  |  | |  |
|  | Ⅰ | 101 | / | 101 | | / |
|  | Ⅱ | 4 | / | 4 | | / |
|  | CEA (0-5 μg/L) | 1.10 (0.70-1.66) | 0.8 (0.65-1.00) | 1.46 (0.90-2.25) | | ＜0.001 |
|  | CYFRA21-1 (0.1-4.0 ng/ml) | 3.95 (3.13-5.05) | 3.25 (2.43-4.81) | 4.12 (3.49-5.21) | | ＜0.001 |
|  | SCC (<1.8 ng/ml) | 1.40 (1.10-1.80) | 1.30 (0.96-1.91) | 1.40 (1.20-1.70) | | 0.168 |
|  | NSE (0-24 ng/ml) | 10.07 (3.82-12.00) | 1.89 (1.08-4.72) | 11.29(9.94-12.68) | | ＜0.001 |
| Validation  cohort（Suzhou） | Sample size | 36 | 19 | 17 | |  |
|  | Age | 56.31±11.91 | 53.21±9.72 | 59.76±13.42 | | 0.100 |
|  | Gender |  |  |  | |  |
|  | Male | 11 | 5 | 6 | | 0.559 |
|  | Female | 25 | 14 | 11 | |  |
|  | BMI | 24.22±2.92 | 24.59±3.03 | 23.82±2.84 | | 0.437 |
|  | Tumor size |  |  |  | |  |
|  | ≤3cm | 15 | / | 15 | | / |
|  | 3-5cm | 2 | / | 2 | | / |
|  | N stage |  |  |  | |  |
|  | N_0_ | 17 | / | 17 | | / |
|  | N_1_ | 0 | / | 0 | | / |
|  | Tumor stage |  |  |  | |  |
|  | Ⅰ | 15 | / | 15 | | / |
|  | Ⅱ | 2 | / | 2 | | / |
|  | CEA (0-5 μg/L) | 1.76(1.19-2.32) | 1.37(0.94-2.00) | 2.24(1.51-2.62) | | 0.018 |
|  | CYFRA21-1 (0-5 ng/ml) | 1.63(0.98-2.43) | 1.12(0.87-1.64) | 2.46(1.72-3.64) | | ＜0.001 |
|  | SCC (<2.0 ng/ml) | 1.23(0.89-1.95) | 0.98(0.89-1.26) | 1.98(0.88-2.40) | | 0.035 |
|  | NSE (0-20 ng/ml) | 4.15(2.45-1.25) | 2.45(2.08-3.09) | 11.30(10.15-12.70) | | ＜0.001 |
| Validation  cohort（Hainan） | Sample size | 27 | 12 | 15 | |  |
|  | Age | 55.41±12.62 | 50.83±14.25 | 59.07±10.19 | | 0.092 |
|  | Gender |  |  |  | |  |
|  | Male | 13 | 7 | 6 | | 0.930 |
|  | Female | 14 | 5 | 9 | |  |
|  | BMI | 24.56±3.02 | 24.93±3.21 | 24.27±2.95 | | 0.581 |
|  | Tumor size |  |  |  | |  |
|  | ≤3cm | 15 | / | 15 | | / |
|  | 3-5cm | 0 | / | 0 | | / |
|  | N stage |  |  |  | |  |
|  | N_0_ | 14 | / | 14 | | / |
|  | N_1_ | 1 | / | 1 | | / |
|  | Tumor stage |  |  |  | |  |
|  | Ⅰ |  | / | 14 | | / |
|  | Ⅱ |  | / | 1 | | / |
|  | CEA (0-5 μg/L) | 1.38(1.01-2.37) | 1.20(0.93-1.87) | 1.96(1.42-2.70) | | 0.055 |
|  | CYFRA21-1 (0.1-4.0 ng/ml) | 1.83(1.23-2.41) | 1.34(1.09-2.21) | 2.30(1.73-3.57) | | 0.019 |
|  | SCC (<1.8 ng/ml) | 0.95(0.77-1.45) | 0.90(0.75-1.13) | 1.16(0.77-1.73) | | 0.351 |
|  | NSE (0-24 ng/ml) | 3.56(2.35-11.80) | 2.45(1.90-3.55) | 13.30(11.60-14.70) | | ＜0.001 |
| Validation  cohort（Beijing） | Sample size | 70 | 26 | 44 | |  |
|  | Age | 55.43±10.85 | 54.35±11.45 | 56.07±10.56 | | 0.153 |
|  | Gender |  |  |  | |  |
|  | Male | 38 | 21 | 17 | | 0.001 |
|  | Female | 32 | 5 | 27 | |  |
|  | BMI | 24.13±2.67 | 24.56±1.55 | 23.87±3.13 | | 0.085 |
|  | Tumor size |  |  |  | |  |
|  | ≤3cm | 44 | / | 44 | | / |
|  | 3-5cm | 0 | / | 0 | | / |
|  | N stage |  |  |  | |  |
|  | N_0_ | 43 | / | 43 | | / |
|  | N_1_ | 1 | / | 1 | | / |
|  | Tumor stage |  |  |  | |  |
|  | Ⅰ | 43 | / | 43 | | / |
|  | Ⅱ | 1 | / | 1 | | / |
|  | CEA (0-5 μg/L) | 1.10(0.80-1.68) | 0.85(0.70-1.10) | 1.27(0.90-2.44) | | 0.003 |
|  | CYFRA21-1 (0.1-4.0 ng/ml) | 3.83(3.07-4.83) | 3.65(2.36-4.79) | 3.84(3.26-4.86) | | 0.319 |
|  | SCC (<1.8 ng/ml) | 1.41(1.20-1.79) | 1.51(1.19-2.10) | 1.40(1.20-1.70) | | 0.243 |
|  | NSE (0-24 ng/ml) | 8.56(2.39-11.72) | 1.87(1.22-4.44) | 10.39(8.59-12.97) | | ＜0.001 |
