# Supplementary material for: Gut mycobiome as a potential non-invasive tool in early detection of lung adenocarcinoma: a cross-sectional study
Source: BMC Med. 2023 Oct 31;21:409. doi: 10.1186/s12916-023-03095-z (PMC10617124; doi:10.1186/s12916-023-03095-z)
Supplement: Supplementary file 2 — Additional file 2. The PCR amplification conditions. Table S3. The first round PCR system. Table S4. The second round PCR system. [file 12916_2023_3095_MOESM2_ESM.docx]

**The PCR amplification condition.**

**Table S3.** The first round PCR system.

| Enzyme Q5 amplification system | 20 µL |
| --- | --- |
| 5 × Q5@ Reaction Buffer | 4 µL |
| 2.5 mM dNTPs | 0.4 µL |
| Primer F (10 µM) | 1 µL |
| Primer R (10 µM) | 1 µL |
| [Q5@ High-Fidelity DNA Polymerase](mailto:Q5@%20High-Fidelity%20DNA%20Polymerase) | 0.1 µL |
| Genomic DNA Template | 4 µL |
| H_2_O | up to 20 µL |

Note: The first round of amplification procedures:

Amplification of genomic DNA in 20-µL PCR reaction mixtures was conducted under the following conditions: 95°C for 5 min; 33 cycles of 95°C for 1 min, 60°C for 1 min, and 56°C for 1 min; followed by 72°C for 7 min.

**Table S4.** The second round PCR system.

| Enzyme Q5 amplification system | 50 µL |
| --- | --- |
| 5×Q5@ Reaction Buffer | 5 µL |
| 2.5mM dNTPs | 1.5 µL |
| 5×Q5@ High GC Enhancer | 1.5 µL |
| Index Primer (10µM） | 1 µL |
| Universal PCR Primer (10µM) | 1 µL |
| [Q5@ High-Fidelity DNA Polymerase](mailto:Q5@%20High-Fidelity%20DNA%20Polymerase) | 1 µL |
| Template | X µL (50ng) |
| H_2_O | Up to 50 µL |

Note: The second round of amplification procedures:

A second round PCR using 50-µL reaction mixtures under the following conditions: 95°C for 5 min, followed by 12 cycles of 95 °C for 1 min, 60 °C for 1 min, 72°C for 1 min; and 72°C for 7 min
