# Supplementary material for: Gut mycobiome as a potential non-invasive tool in early detection of lung adenocarcinoma: a cross-sectional study
Source: BMC Med. 2023 Oct 31;21:409. doi: 10.1186/s12916-023-03095-z (PMC10617124; doi:10.1186/s12916-023-03095-z)
Supplement: Supplementary file 3 — Additional file 3. Supplementary figures. Fig. S1. Abundance differences of the 19 OTUs between the LUAD and HC groups in the Beijing discovery cohort. All of the 19 OTUs are significant different in the two groups (Wilcoxon rank-sum test, the respective P-values are shown in the diagram). Fig. S2. AUC of the selected OTU features for LUAD validated in the matching cohort. [file 12916_2023_3095_MOESM3_ESM.docx]

**Supplementary figures.**

**
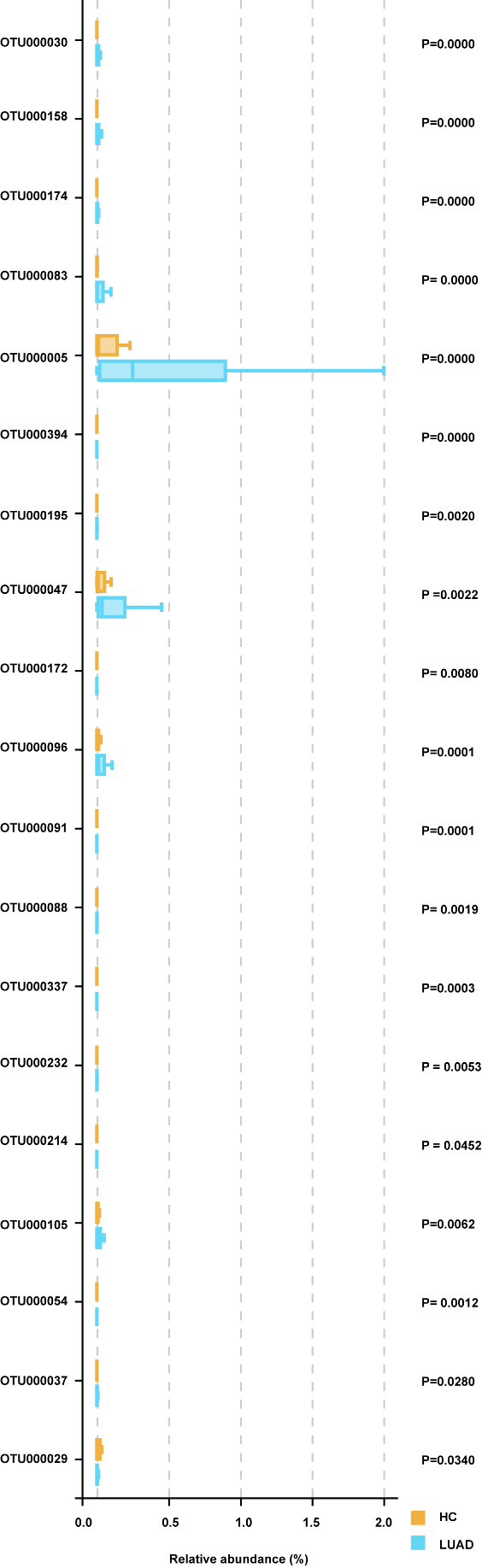
Fig. S1**

**Fig. S1.** Abundance differences of the 19 OTUs between the LUAD and HC groups in the Beijing discovery cohort. All of the 19 OTUs are significant different in the two groups (Wilcoxon rank-sum test, the actual P-values are shown in the diagram).

**Fig. S2**


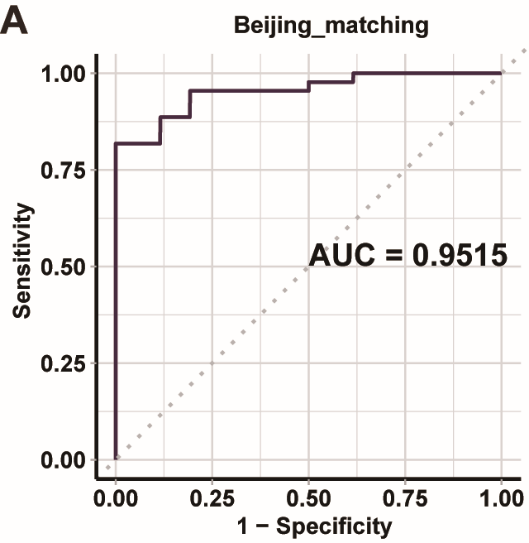


**Fig. S2.** AUC of the selected OTU features for LUAD validated in the matching cohort.
